# Supplementary material for: A Qualitative Study Examining the Illness Narrative Master Plots of People with Head and Neck Cancer
Source: Behav Sci (Basel). 2019 Oct 17;9(10):110. doi: 10.3390/bs9100110 (PMC6826984; doi:10.3390/bs9100110)
Supplement: Supplementary file 1 [file behavsci-09-00110-s001.pdf]

Supplementary File

This file considers the stages of development for the analysis of the paper. Examples are given for each stage. The fully analysis is available from the corresponding author.

**Appendix A**
Example of the first stage of the analysis

**Example 1**

**1. Thoughtful and Responsible or Reflective and Fatalistic.**

**What is the basic plot to this story?**
The basic plot is that that life is fragile and there are inevitabilities. Details and realities are described through past experiences of loss and explain the context of how hard it can be. Accessibility of knowledge to adapt to the approaching decline, explains the use of selected experts who are valued. Ways to cope are described with the conclusion in this plot that the best has been done in the circumstances.

**What are the defining elements of the main plot?**
This story shines light and brings detail to the reality of life. There is awareness of the sudden changes and the associated losses that occur explained in objective rather than emotive terms. There are different methods of coping detailed from the initial premise of one's own mortality. The goal within this plot is to be as well as possible despite the reality of the diagnosis and treatments. The story refers back to examples relating to mortality, or the threat of it from close family, fatalism is understood.

"*Of course it's ridiculous (looking at the statement its difficult to contemplate your own mortality) you have to think about that anyway we are all going to get there. I've always been a realist I always thought if anything should happen to me this is what I want as opposed to I won't think because it will tempt fate.*" 9.18-9.28 KG

**How does the teller view themselves now?**
There was always the desire to be treated and although the reality might be difficult there are no regrets in having chosen treatment. Pre-morbid activities, including work, are returned to and although this might be in a different guise the structure or form of normality is sought.

**Does this narrative consider or use acceptance of what has happened?**
There is acceptance of a change in function and that there may not always be good news when attending outpatient appointments. There is also an acceptance that the diagnosis brings about change, which does not have to be understood by others because they are not affected by it.

**How does the story consider treatment and hope?**
The story considers that if treatment was offered there was no real choice but to have it. Through achieving the goal of being alive emotions and uncertainty are bypassed at times in order to limit worry.

**What does the story embrace or not allow?** The story shows a detachment recognising that individuals react differently to the situation. A rational stance also explains why presumptions are not made to give specific advice to others who have cancer. There is insight that within the social network people are unlikely to be effected significantly by the news of the disease, which is acknowledged as a reaction that the teller would have had in a similar scenario. There is no desire to seek to be the centre of attention following the initial diagnosis. Acquaintances are told on a need to know basis and the amount and content that is disclosed is modified.

**How does the story deal with symptoms?**

There is evidence of resilience with a sense of reality and humour in descriptions of symptoms. The experience is coped with more easily by tasks and periods of time being broken-down into smaller units. There is information relating to the recovery, which details how much slower it is than expected leading to frustrations. In this story no treatment is off limits as long as it gives the maximum possible outcome.

**How does the story use interaction and treatment?**
Skilled communication techniques are used in order to help make judgements about the HCPs that are met. Relationships are only built with those perceived by the teller to be beneficial. Key HCPs are used to develop conversations about the treatment impacts from the belief that people respond as an individual. It is unlikely external information from either the Internet or written literature will be referred to because there is a perception that details are too general, instead a reliance on chosen HCPs is preferred.
*"Your imagination is running riot and what you need is the parameter that says "but that's not even on the cards because you aren't in that ballpark"*. It's so helpful. You don't know and you need to talk to someone who can say *"hang on let's go with the reality here"*. *If you read on websites you don't know how much relates to you …..you need that quality input, guidance from what to expect now or in the next few steps*". 6.17-7-.12KG

There is recognition that some HCPs have advanced technical skills and knowledge. When HCPs are met a decision is made about whether a trusting relationship can be built. The tellers define for themselves the direction of recovery and the parameters they want to make judgements on. Chosen HCPs are sought to support and understand the rate of recovery. The story does not want false-hope from HCPs because there is an acceptance that HCPs cannot just impart good news.

*"You might not want to believe it but that's different from not having it explained to* you" 1.20-1.21 CF.

**How does the story consider hope and recovery?**
The reality of a possible decline or permanent symptoms post-treatment is acknowledged and the tellers of this plot are aligned to reality rather than hope.

**Are there any anti-narrative?**
There is a sense of distance and objectivity recognising the space needed for patients to react as individuals.
"Everyone copes with it in their own way, drama queens versus bluffers" 2.40 CF

**Appendix B**
An example of the second stage of the analysis can below.

**<u>Illness Narrative -Change and Recovery:</u>**

**The story:** -A subset of Quest
Nothing is like the reality of being treated. They are not surprised they are diagnosed with cancer. They develop a relationship that is productive for them with a small number of the team knowing that there are key people to relate to.
They pace themselves through key times of the treatment recognising that the treatment was gruelling and very hard to cope with. They use strategies such as braking tasks into smaller stages in order to still take part in family activities, knowing that they function differently. They have insight that they are not able to manage as they did prior to their disease, but they know they have to carry on and acknowledge how well they are in the context of such treatments and disease.

**Choice in decision to be treated:**
When they knew something could be done that was all that mattered. Not having treatment was never an option. There are no regrets, or doubt about the choice and they refute strongly that they wish they had never been treated.

**Goals of the patient:**
Cure is the most important thing. Being cured of cancer is the overriding goal. They set themselves achievable end-points to measure personal progression. Goals and resources are set in the present, not too far ahead and split into small stages, which in their terms made them more achievable. The participants could reflect back further, but kept forward look on closer horizons.

*"I can see there's change; like I've had a tin of biscuits and I've tried one. I thought I won't push that I might have 2 next time" GM 3.31-3.32*

**View of embracing physical; psychological and spiritual wellbeing:**
They had considered themselves as ill and were recovered but acknowledged the extensive time-frame and challenge it had been to accomplish this.

*"The last time I was in hospital was 18th October and I thought there was a possibility of going back to work before Easter in the April. Even then … I thought once I'm off that tube (Nasogatric tube) I'll be up and away and off I'll go; and the first meeting I had about going back to work was after Easter."*
Sort number 6: 1.34-1.40

The experience and diagnosis had not meant they talked about different topics.
There was no sense of feeling isolated physically or emotionally and they did not feel exhausted physically. There were some feelings of vulnerability in hospital but at home they were not frightened although they had feelings of vulnerability at home.

*"We both dreaded (patient and partner) the weekend at home. When I had the feeding tube (NGT) in, it was horrendous we would 'phone the ward, they were brilliant and whoever was the duty-registrar would say "Bring her in" even if it was only for 2 days….We felt very vulnerable particularly when it blocked I tried with fortisips and during this time the weight just fell-off me, I never noticed and you are not wearing the same clothes. You became so vulnerable to a little tube blocking up…that's the only time we read the information - tricks to unblock the tube …one time …….Halleluiah." EN 4.1-4.32*

Pain was not a major symptom but that was because they were aware it was being managed at different points during the treatment.
They disagreed strongly that people stared at them. If they did they were not concerned about that aspect and were ambivalent about the effect the treatment and disease had on their appearance.

**Interaction with HCPs and the information they are given.**
There was insight into how many people they met over the course of diagnosis and treatment. Despite the numbers they could remember the key people.

*"Obviously surgeon, oncologist but on a personal level my GP was brilliant and the speech therapist was great and one of the ward nurses and the district nurses..not all the nurses were great. So of a team of about 90 people about 4 or 5 and, you can narrow that down further to those who had the most impact. …. Some people are just filling in their forms- it's a process "I've done my bit" –like a sausage factory……… if only you could be seen by 30 people in a better way than 90 doing a half-baked job of it." EN 9.35-10.36*

They would remember discussion with the team who they felt treated them as an individual. They did not write anything down or read any literature they relied on building relationships with key-people in the team to create conversations. They will not research the disease and treatments because they know that people have to be understood at their individual level. They relied on the MDT for information within the consultation to support them with their next treatment, or review goals.

*"I never read the information. I guess I didn't want to know …….my wife read up on it I just did not want to know, it was as if the detail didn't bother me I wanted to know a little bit but when they give you a book that thick on neck dissection that was of no interest"*
JH: 1.1-1.7

*"We hung on every word my partner, my sister, my brother, we are from the old school, the consultants are God, and you bow down to everything they say. He (the surgeon) was ultra ultra professional ……bit distant really is that their job to be distant so that other people can be more touchy feely or whatever?"*
EN: 9.4-910

They were not prepared for the changes that were the reality in the post-treatment phase. One of the participants commented on the impact of the treatments by saying
*"No, [current presentation] is nothing like the reality the long-term effects are not particularly explained the fact I am numb from the top of my ear, puffy round the neck ……….that was never really explained……'*
JH: 1.10-1.14

They were sensitive to non-verbal interactions of the team and would pick up on signals, or the lack of them. The non-verbal aspect during an assessment could make them feel vulnerable.

*"I'd upset them (the imaging team) I was on an appointments list and the list had obviously slowed down and I knew that as opposed to do an ultrasound only they were now going to do a biopsy too. I knew that was all going on I could feel it and then when they were actually looking on the screen and pointing they didn't handle me very well what so ever ……..at one point there were 3,4, 5 people in a small room -it's very enclosed. That's when you feel alone you feel like saying "Hello I'm here. Talk to me" ……but of course they weren't they were talking to one another and muttering and as I say there was quite a lot of politics going on because I picked up they had not got enough of the sample in outpatients so they were needing to repeat it there with the imaging." E.N 4.2-4.31*

They did not feel extended members of the team treated them as an individual. They felt able to build meaningful and trusting relationships with key members of the team, which meant they could work through discrepancies with them. They would know that no matter how busy a professional appeared they should take opportunities to discuss specific topics they wanted to because it would help them adjust or understand their current situation. As this was their main channel of information they were able to use it as they needed to with chosen HCPs.

**Coping and Psychological Adaptation**
They display tenacity and stoicism as a way of coping. No matter how bad it was they would cope. There was no apparent emotional cost in terms of being more irritable as a consequence of the experience in fact the participants disagreed that they were any more irritable as a result of the experience. They seem always to have an end-point in their sights, which is encouraging them through the hard times. A reality check is applied of it could not possibly be easy; it was treatment to the head and neck over a protracted time.

*"That horrible mask…. I got through it with will power I had to have the treatment to have the treatment I knew I had to wear the mask and that's that.'*
GM 4.33-5.3

They had not understood the reality of the treatment until it was experienced.

*"You don't understand that however many doses of Radiotherapy it is……. 30 that this will mean all of that when you don't know all of that. You think OK neck dissection August…… treatment mid-September, October, November Oh I might be back at work in December …..I was so stupid I had seen my mother have cancer right in the middle of her head in the late 1980's……I still did not put that together with mine. I remember saying to people "I've got this radiotherapy" but I thought operation, including teeth out (tonsillectomy, neck dissection) radiotherapy back at work. I didn't see any of the other experiences just the landmarks. They didn't have any real meaning until you were in your nighty feeling utterly exhausted…… feeling very tearful"* EN 2.5- 2.26

The coping was through breaking down events into stages.

*"I never thought of myself as brave you deal with it in the bits that you can. For the radiotherapy I got the timing sussed. Knew the sounds to listen for this sound and that sound and I could work out how long 'til it stopped." EN 12.23-12.32.*

The small amounts and little stages continue post-treatment.

*"Going out for a meal I have soup of the day with no roll. The family tuck into a full meal and I try bits off their plates and part of me would love to have what they have. There's enough people there who can cover fro me and my daughter is very protective." GM 1.20-1.26*

*They appear to have inner resources that are strong.*
*"You make your bed and lie on it ..you do something you sort it out, it makes you stronger and independent some things go wrong, and you sort it out" GM 3.33-3.38*

**View of progress:**
Participants identified strongly with being ill, and believed their recovery was much slower than they anticipated, finding it hard waiting to heal. They were surprised and pleased with themselves at their ability to adjust physically. They acknowledged that the process was awful but in their recovery they were not preoccupied by pervasive symptoms.

*"Tomorrow you might be a little bit better……you don't realise that until someone says to you "you've done so and so". It's not day-on-day but month-on-month." GM 2.29-3.30*

**Acceptance and embracing of hope:**
Life would never be the same again but they would deal with the treatment because it was being offered as an option. There was no particular belief that they had become grateful for different things in life.
They continue with some activities but in different ways. Aware of adjustments they have made and wondering about how close to their previous routines they might get.

"We used to go to the theatre and go for a Cantonese ..I went to the pantomime with my son and his two children I really enjoyed it. I got my water and lovely toilets…….I can do some of this …. It's got me thinking. My son bought me a coffee with extra milk and I sat in one of the restaurants and I really enjoyed returning to normal life ………it's just when will I go to a restaurant and ever have a proper meal…….?" GM 3.6-3.16

**View of Mortality:**
They had always felt it was possible that they would be diagnosed with cancer; they had had close family members who had died of the disease. They found it hard to contemplate their own death and had not ever thought of their own mortality prior to the diagnosis.

*"I always thought it could happen to me. Both my parents died of cancer so I'd be stupid not to think that after the CT scan."*
EN: 2.33-2.35

Any new symptoms after treatment that they experienced might be recurrent disease. They are pragmatic if this were so they would need to remain engaged with the systems and talk through with the team possible options rather than worry unduly.

Appendix C The reduction of content towards a common plot

**<u>Illness Narrative –The Recovery Narrative:</u>**

**The story:** -A subset of Quest?
The basic plot is to this story is that one can understand and respond to the reality of the illness in an effective way. To manage the symptoms and effects of the illness is to overcome and continue in life. The symptoms and problems created by the illness are view as a hurdle, examined for what they are but consider as something which can be overcome. The story is defined by taking action in order to continue desires and life in an acceptable way, to do nothing is never an option, the story doesn't allow for shock or surprise or account for losses because it is focused on what can be changed in the present in order to manage and benefit the future. This story embraces change and loss and is accepting to the diagnosis and even the potential of mortality (past experiences of the illness with other family members may aid this). The embracement of change appeared to accompanied by the need to act and utilise past coping skills. These skills provide a framework to achieve a goal (such as visiting a friend or getting back to work) through problem solving. For instance, one patient stated;

> *"The last time I was in hospital was 18th October and I thought there was a possibility of going back to work before Easter in the April. Even then … I thought once I'm off that tube (Nasogatric tube) I'll be up and away and off I'll go; and the first meeting I had about going back to work was after Easter."*
> Sort number 6: 1.34-1.40

The teller does not deny the difficulties faced and expresses that to live through treatment they will need to break task down and approach them in different ways. The teller is able to recognise time of vulnerability, but still would emphasise the ability to overcome. For instance, one participant stated:

> *"We both dreaded (patient and partner) the weekend at home. When I had the feeding tube (NGT) in, it was horrendous we would 'phone the ward, they were brilliant and whoever was the duty-registrar would say "Bring her in" even if it was only for 2 days….We felt very vulnerable particularly when it blocked I tried with fortisips and during this time the weight just fell-off me, I never noticed and you are not wearing the same clothes. You became so vulnerable to a little tube blocking up…that's the only time we read the information - tricks to unblock the tube …one time …….Halleluiah." EN 4.1-4.32*

The teller can see a time point in the future where, through challenge, recovery will have been gained. The action taken is never doubted because to doubt it would be to look backwards something which this particular story denies.

The teller of this story often uses interaction and action with health care professions to aid and enhance the action taken and the choices made for treatment. Individuals appeared to develop and invest in trusted relationships, where they perceived the health care professional was invested in them and with this the HCP was deemed effective, for instance one individual commented;

> *So of a team of about 90 people about 4 or 5 and, you can narrow that down further to those who had the most impact. …. Some people are just filling in their forms- it's a process "I've done my bit" –like a sausage factory……… if only you could be seen by 30 people in a better way than 90 doing a half-baked job of it." EN 9.35-10.36*

The danger of this requirement in relationships appeared to be that they were sensitive if the interaction lacked a standard expected, for instance an individual stated; *"at one point there were 3,4, 5 people in a small room -it's very enclosed. That's when you feel alone you feel like saying "Hello I'm here. Talk to me" E.N 4.2-4.31.*

Further to this it appeared to be that they may not question the advice given or seek understanding in other ways leading to unexpected changes, for instance one individual commented;

> *"No, [current presentation] is nothing like the reality the long-term effects are not particularly explained the fact I am numb from the top of my ear, puffy round the neck ……….that was never really explained……'*
>
> JH: 1.10-1.14
